# Supplementary material for: Neuronal parts list and wiring diagram for a visual system
Source: Nature. 2024 Oct 2;634(8032):166–80. doi: 10.1038/s41586-024-07981-1 (PMC11446827; doi:10.1038/s41586-024-07981-1)

Tm32

predicate precision: 85%, recall: 89%

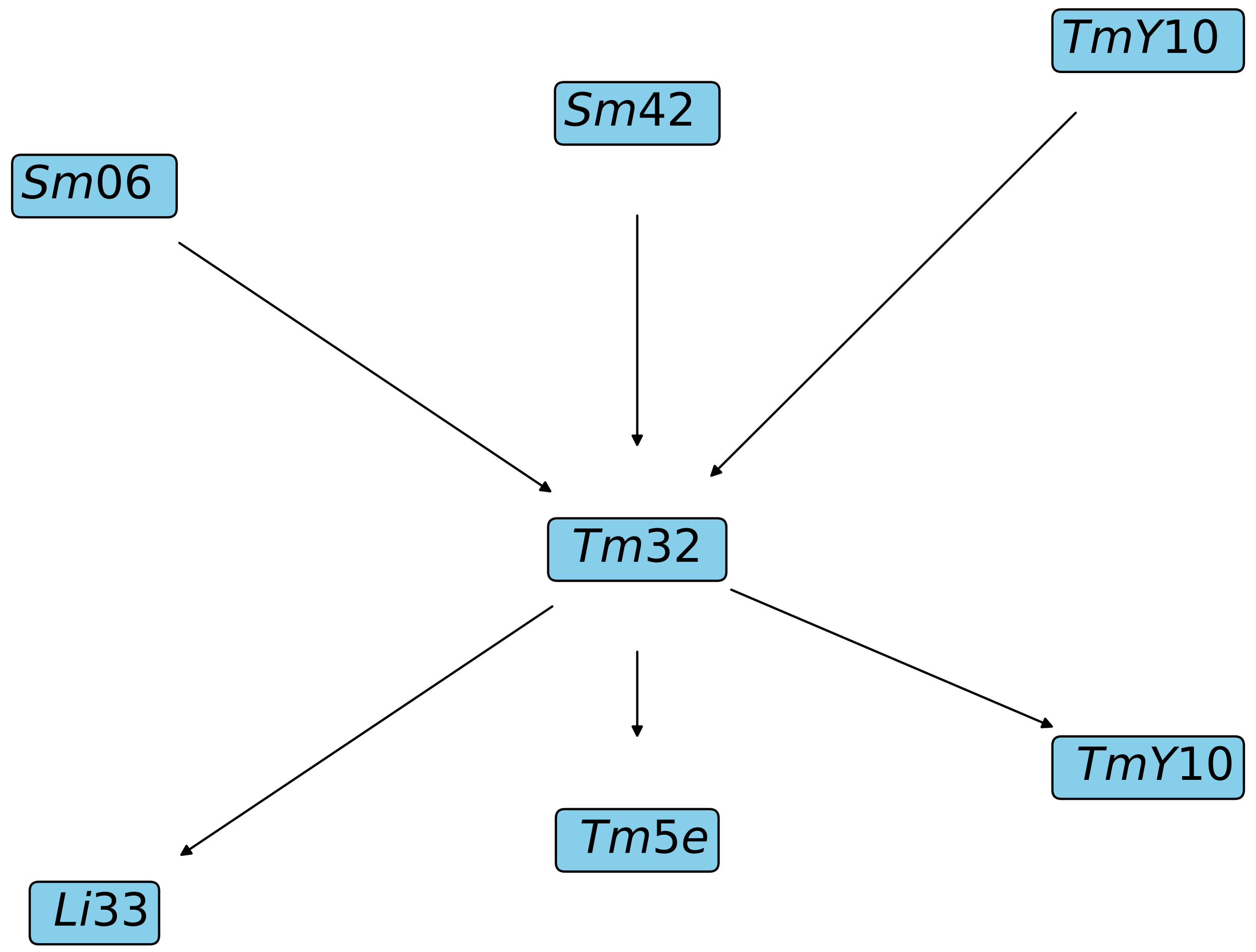

avg. stats

cells	76
NT prediction	GLUT (96%)
top in/out regions	ME (59%) / LO (70%)
in/out degree	42 / 43
in/out synapses	174 / 161
cable len	667μ
surface	963μ^2
volume	58μ^3

morphology

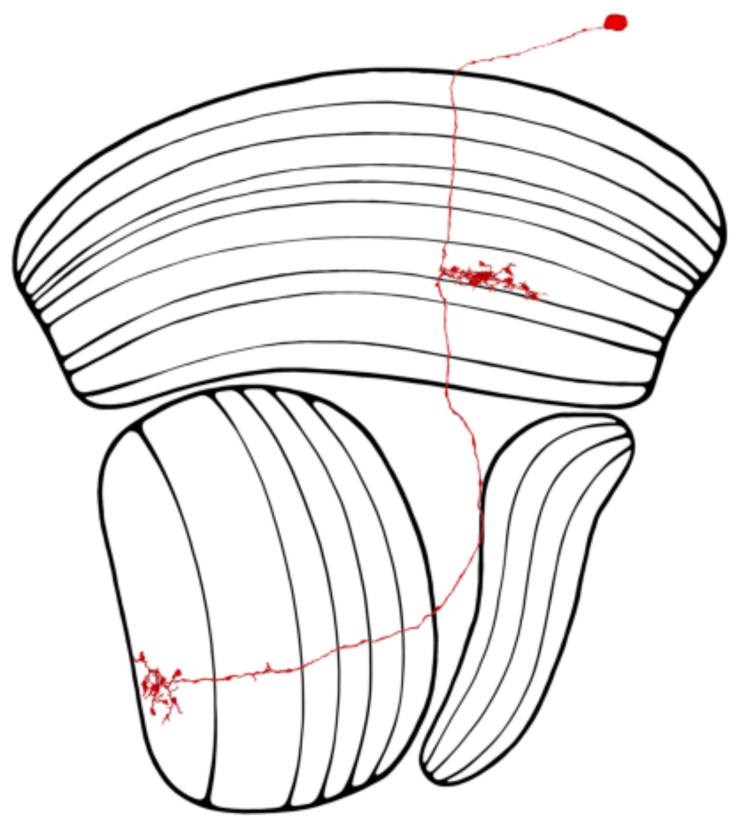

anterior view

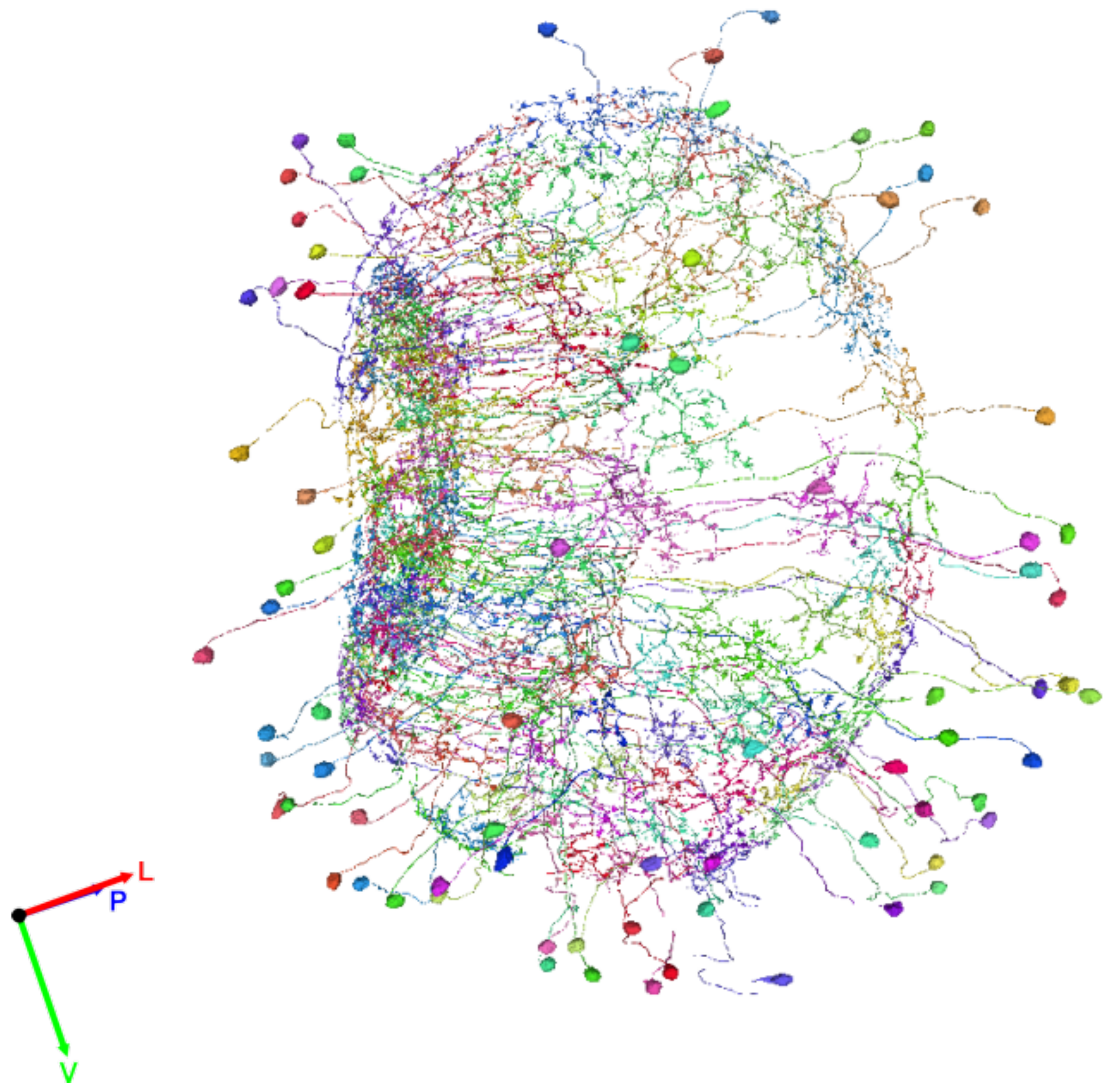

ventral view

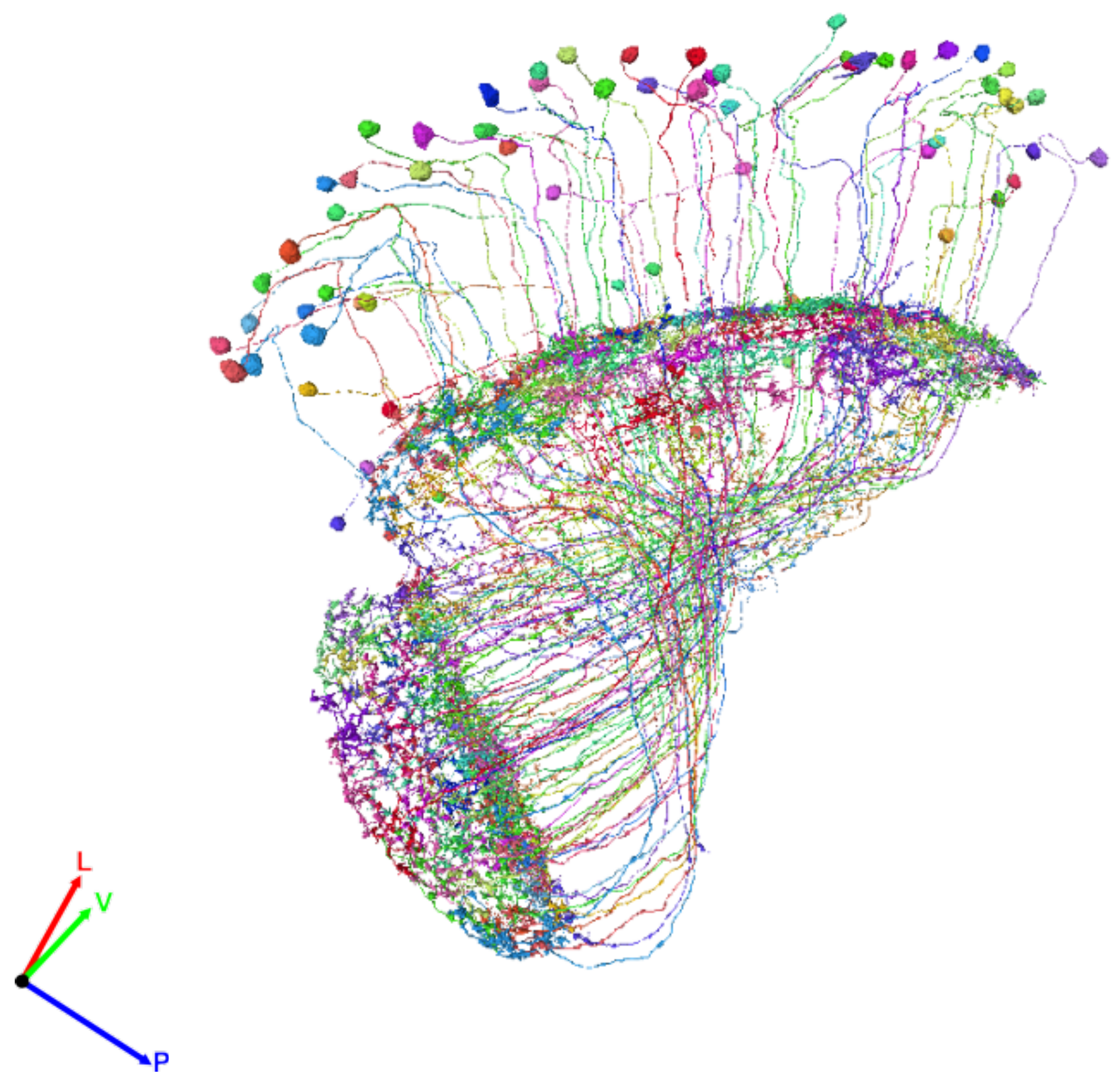

side view

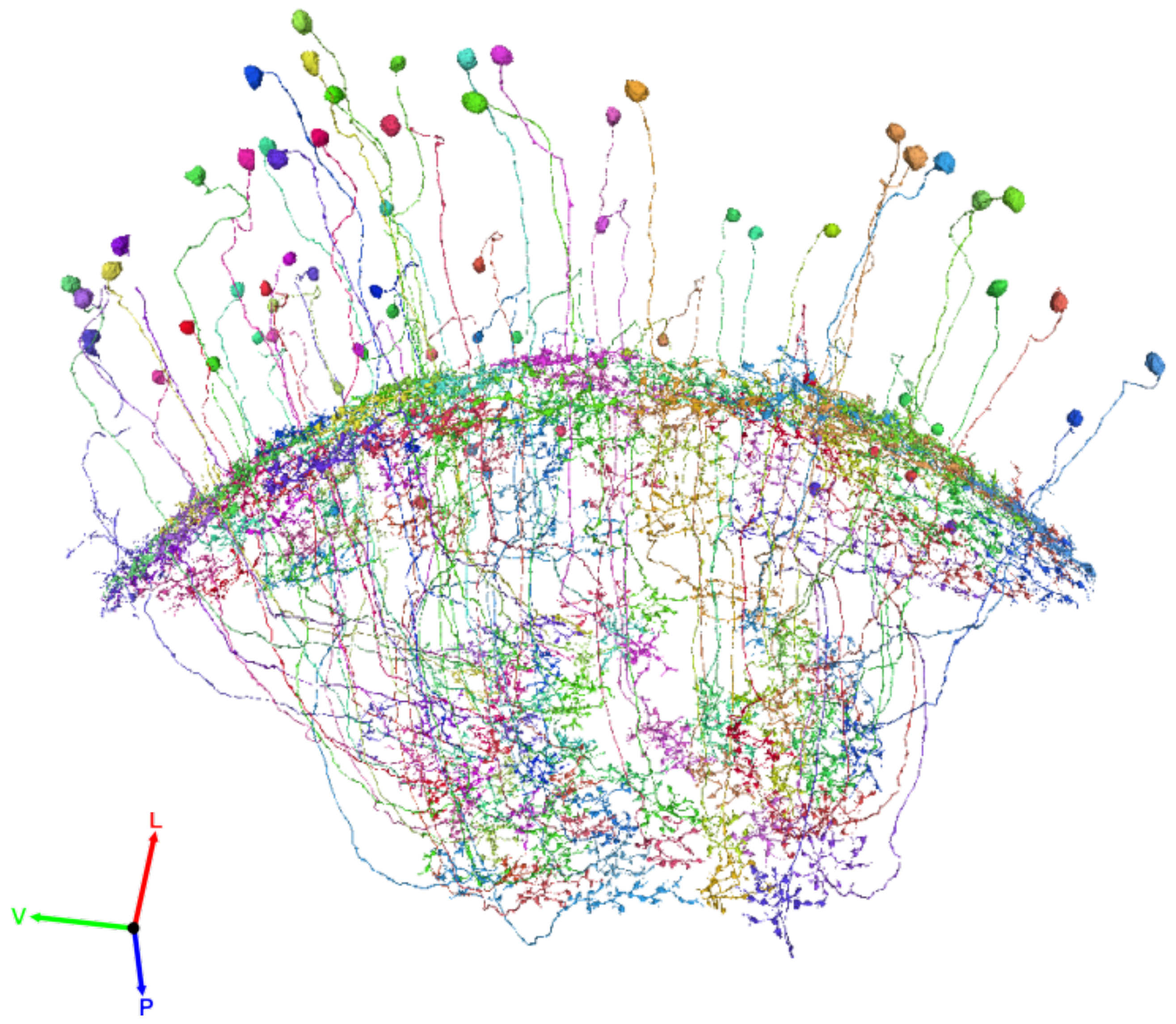

Supplement: Supplementary file 5 — Cards for all cell types. Each card has 6 panels (clockwise): logical connectivity predicate diagram, numeric stats, illustration of a typical cell, followed by 3D rendering of all cells from three orthogonal viewpoints. Also available at Codex. [file 41586_2024_7981_MOESM5_ESM.zip › pdf/Tm32.pdf]
